# Measurement of In-Plane Motions in MEMS

**DOI:** 10.3390/s20123594

**Published:** 2020-06-25

**Authors:** Mohamed Arabi, Mary Gopanchuk, Eihab Abdel-Rahman, Mustafa Yavuz

**Affiliations:** 1 Department of Systems Design Engineering, University of Waterloo, Waterloo N2L 3G1, ON, Canada; eihab@uwaterloo.ca; 2 Design & Engineering, Polytechnic University of Milan, Piazza Leonardo Da Vinci, 32, 20133 Milano MI, Italy; maryolena@hotmail.com; 3 Department of Mechanical and Mechatronics Engineering, University of Waterloo, Waterloo N2L 3G1, ON, Canada; myavuz@uwaterloo.ca

**Keywords:** measurement, in-plane, MEMS, LDV, comb-drive

## Abstract

We report a technique to measure in-plane and out-of-plane motions of MEMS using typical out-of-plane (single-axis) Laser Doppler Vibrometers (LDVs). The efficacy of the technique is demonstrated by evaluating the in-plane and out-of-plane modal response and frequency response of an interdigitated comb-drive actuator. We also investigate the validity of observing planar modes of vibration outside their dominant plane of motion and find that it leads to erroneous results. Planar modes must be evaluated in their plan of motion.

## 1. Introduction

The dimensions of microelectromechanical systems (MEMS) preclude the use of traditional inertial sensors, such as accelerometers, to measure their motions. Although electric parameters, such as capacitance [1] and current [2], can be employed to indirectly estimate the motions of MEMS, they have limitations. To begin with, separating features in the measured signal due to motions from those due to electric circuit interactions is nontrivial. Even where that is possible, the measured signal represents an ‘integral-type’ estimate of the overall structure motion rather than a ‘point-wise’ measurement of motion. As a result, the gold-standard for MEMS motion measurements has been non-contact optical techniques, such as Laser Doppler Vibrometry (LDV) [3] and digital image correlation (DIC) [4,5].

**Figure 1:**
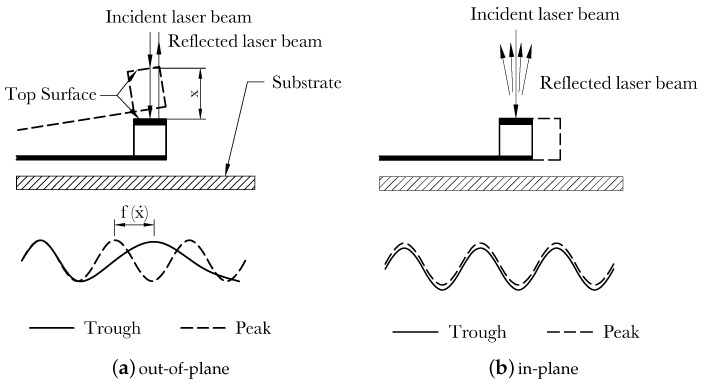
Working principle of LDV.

Typical LDVs detect out-of-plane motions by measuring the Doppler effect, the difference between the frequencies of the incident and reflected laser beams from a point traveling along the beam propagation direction [4,6], Figure 1a. They cannot detect in-plane motions since they do not result in a Doppler effect, Figure 1b. Various techniques have been proposed to overcome this limitation and enable LDV measurement of the in-plane motions. They can be classified into on-axis and off-axis techniques [7].

On-axis approaches place the incident laser beam and the photodetector receiving the reflected beam along an axis aligned with in-plane motions [7]. The main drawback of this technique is the complexity of the experimental setup required to access the sidewalls of in-plane moving MEMS. Furthermore, the lack of an objective lens makes this approach feasible only for larger MEMS. Turner et al. [8] overcame these limitations by fabricating a $45^{\circ }$ micromirror on the same substrate as the MEMS under-test to gain optical access to the sidewalls using a single-axis LDV. This approach requires significant fabrication capabilities not available in many standard processes. It also requires an addition footprint to equip each MEMS under-test with a dedicated micromirror.

Off-axis approaches use photodetectors placed at an angle with respect to the incident laser beam. In 3D LDV [9,10], an incident laser beam is directed at an angle with respect to the top surface of a moving MEMS. One photodetector is placed on-axis, to measure out-of-plane motion, while two photodetectors are placed off-axis to measure in-plane motions [10,11]. This method allows for simultaneous measurement of in-plane and out-of-plane motions. However, it is rather an expensive technique and the experimental setup is inflexible requiring the capture of reflected laser beams by all three photodetectors. Furthermore, the field of view and the maximum measurable displacements are limited.

A more complex off-axis approach used for larger structures deploys three single-axis LDVs (3-LDV) at different angles with respect to a common surface to measure three velocity vectors and reconstruct its three-dimensional motions out of them [12,13,14]. The main drawbacks of this technique are optical cross-talk between the three laser beams [11] which is hard to avoid at microscale in addition to complexity and cost.

Reu et al. [14] compared the use of DIC and 3-LDV to measure the vibrations of a 7${}^{\prime \prime }$ × 7${}^{\prime \prime }$ plate. They found that the initial experimental setup was laborious in both cases requiring two hours to place and calibrate the LDVs and cameras. The time-scale for data acquisition and analysis was also on the order of hours in both cases. They also concluded that the accuracy of DIC and 3-LDV were similar for in-plane motions. The only differences were the lower cost of DIC and the higher bandwidth of 3-LDV.

Therefore, it is desirable to develop simple methods to measure in-plane motions using single-axis LDV. In this paper, we demonstrate a simple and flexible on-axis method to measure in-plane motions using a standard single-axis LDV.

## 2. Methods

**Figure 2:**
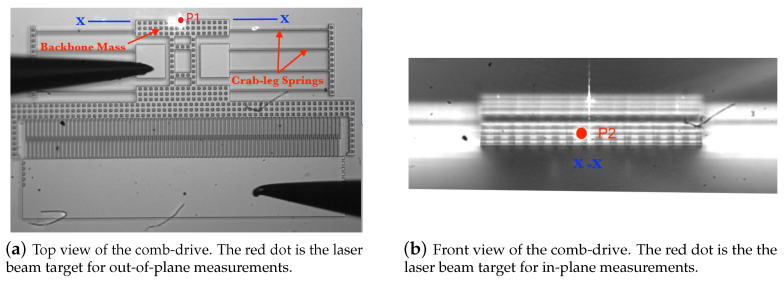
Top and front views of the actuator.

The device-under-test (DUT) is a 59-finger comb-drive actuator fabricated using a single mask Silicon on Insulator (SOI) fabrication process [15] out of a 20 µm device layer. The backbone mass is connected via crab-leg springs to posts on either side of the center-line, Figure 2. Each crab-leg spring is made of four beams 250 µm long, and 3 µm wide.

Typically, LDVs detect the change in frequency between the incident and reflected laser beams from a spot on a surface to measure the out-of-plane velocity of the surface at that point. MEMS moving in-plane, do not produce a frequency change and, therefore, their in-plane velocity cannot be measured. In principle, an LDV can be used to measure in-plane motions by rotating the substrate 90° to make sidewall motions appear out-of-plane with respect to the incident laser beam. However, this requires a clear line-of-sight to the moving wall, unobstructed by other MEMS or anchors on the substrate. Instead, we demonstrate a more practical method to measure in-plane motions regardless of the availability of a clear line-of-sight.

**Figure 3:**
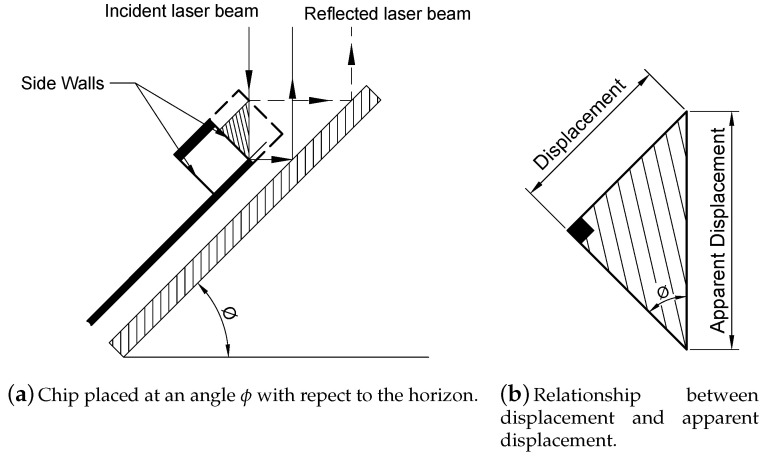
Measurement of in-plane motions.

A stage was designed and fabricated to hold the MEMS chip at an arbitrary angle with respect to the incident laser beam as shown schematically in Figure 3a. The incident laser beam on the moving wall is reflected to the substrate which in turn reflects it back to the objective lens. The relationship between the in-plane displacement and the apparent displacement, Figure 3b, measured by the LDV can be described as:

(1) Displacement=apparentdisplacement×sinϕ

where ϕ is the tilt angle of the stage.

**Figure 4:**
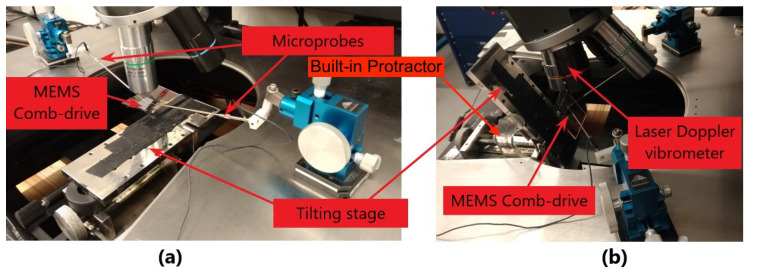
Experimental setup for (**a**) out-of-plane and (**b**) in-plane measurements.

The point marked P1 in Figure 2a is the laser beam target used to measure out-of-plane motions. It is located on the top surface of the mid-point along the back edge of the backbone marked X-X in Figure 2a. In-plane motions are unobservable at this point. The incident laser beam is focused at point P2 to observe in-plane motions, Figure 2b. It is located at the mid-point of the back edge’s side wall, right below point P1. To gain access to point P2, the chip was tilted $70^{\circ }$ with respect to the horizon as shown in Figure 4b. Similar to the previous case, out-of-plane motions are unobservable at this point. The tilt stage was equipped with a built-in protractor to accurately measures the tilt angle and held it constant throughout the experiment as shown in Figure 4.

## 3. Results

LDVs provide a point-wise measurement of motions at a specific point rather than over surface. To capture the surface deformation of a structure at a natural frequency, the mode shape, a multi-point scan is typically carried out over the surface. This is a time-consuming process. In addition, typical LDVs only capture a planar projection of 3D modes. This shortcoming can be remedied by deploying FEM or other simulation tools to carry out modal analysis of the DUT in order to guide us to the modes of interest, to the relevant surface to scan for each mode and to reveal the full 3D mode shape.

**Figure 5:**
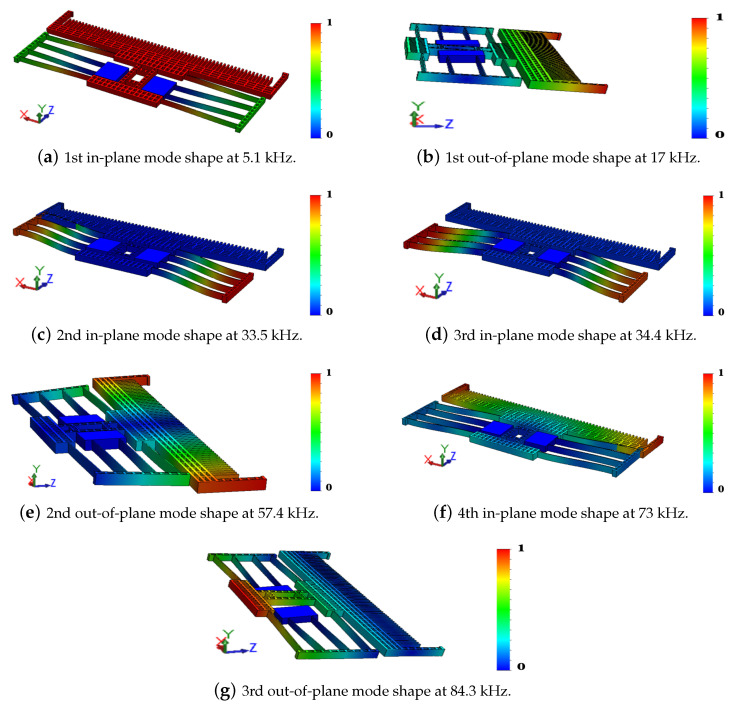
Actuator mode shapes.

Modal analysis was carried out in SolidWorks on a FEM of the actuator to identify the modes of interest. The first seven natural frequencies were found to correspond to four in-plane and three out-of-plane modes. The fundamental mode is in-plane appearing at a natural frequency of $f_{i,1}\;=\;5.1$ kHz where the actuator behaves as a longitudinal comb drive with the backbone mass translating along the z-axis, Figure 5a. In the second and third in-plane modes, $f_{i,2}\;=\;33.5$ kHz and $f_{i,3}\;=\;34.4$ kHz, the crab-leg springs move out-of-phase and in-phase, respectively, while the backbone mass is almost stationary, Figure 5c,d. In the fourth in-plane mode, $f_{i,4}\;=\;73$ kHz, the springs and backbone mass move relative to the posts, Figure 5f.

In the first and second out-of-plane modes, $f_{o,1}\;=\;17$ kHz and $f_{o,2}\;=\;57.4$ kHz, the backbone mass rotates about the x-axis and z-axis, Figure 5b,e, respectively. In the third out-of-plane mode ($f_{o,3}\;=\;84.3$ kHz), the crab-leg springs bend out-of-plane along the y-axis. Simultaneously, the rotor-side of the comb finger rotates around the x-axis to move in the same direction. Therefore, only the first in-plane bending mode and the third out-of-plane bending mode produce significant backbone motions necessary for actuation and sensing purposes.

**Figure 6:**
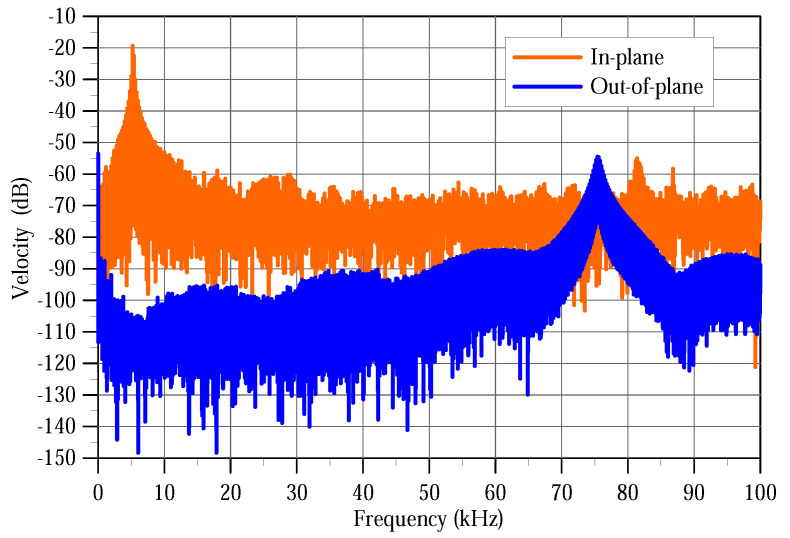
FFT of the measured out-of-plane and in-plane velocities of the back edge under a pulse train of a 75 V amplitude, 200 Hz frequency, and 1% duty cycle.

We obtained the out-of-plane and in-plane modal responses of the backbone by observing the velocity of points $P_{1}$ and $P_{2}$, respectively, under a train of 75 V pulses with a frequency of 200 Hz and a duty cycle of 1%. The Fast Fourier Transforms (FFT) of the measured velocities over 12 periods are shown in Figure 6 in blue for point $P_{1}$ and orange for point $P_{2}$. The power spectrum is presented in dB-scale where 0 dB corresponds to 1 mm/s.

The first peak at $f_{i,1}\;=\;5.1$ kHz, observed in in-plane motions only, corresponds to the first in-plane mode shown in Figure 5a. The second peak at $f_{o,3}\;=\;75.5$ kHz, observed in out-of-plane motions only, corresponds to the third out-of-plane mode shape as shown in Figure 5g. We note that the velocity amplitude of the first in-plane mode 108 mm/s is much higher than that of the third out-of-plane mode 1.8 mm/s. A third peak was observed in-plane at 81 kHz corresponding to a coupling between the two active modes. The noise floor of the in-plane motions was higher than that of the out-of-plane motions at about −50 dB compared to −20 dB, respectively. This is due to the lower intensity of the reflected laser beam in in-plane measurements compared to that in out-of-plane measurements.

The frequency-response of the comb-drive was found by grounding the rotor and applying a voltage signal to the stator

(2) $$V\left(t\right)=V_{o}+V_{o}\cos\left(\Omega t\right)$$

where $V_{o}$ is the signal bias, amplitude, and Ω is the signal frequency. The LDV was used to record the time-history of the out-of-plane (point $P_{1}$) and the in-plane (point $P_{2}$) velocities as the signal frequency was swept. Frequency-response curves were constructed by evaluating the Root Mean Square (RMS) of the velocity over windows of 100 signal periods as a function of frequency.

**Figure 7:**
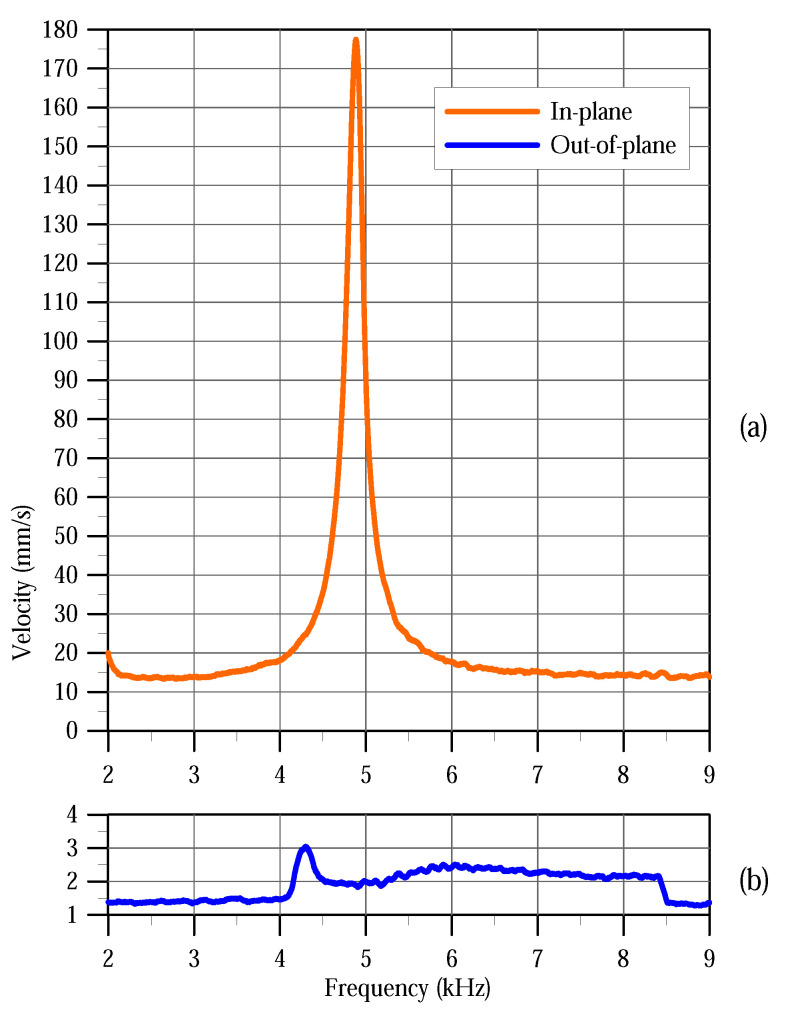
Frequency-response curves of the actuator under the excitation waveform $V_{o}=7.5$ V as the frequency is swept from 2 kHz to 9 kHz. (**a**) in-plane measurements, and (**b**) out-of-plane measurements.

Figure 7 shows the out-of-plane and in-plane frequency-response curves of the actuator under the excitation waveform $V_{o}=7.5$ V as the frequency is swept from 2 kHz to 9 kHz. The peak of the in-plane frequency-response curve appears at $f_{i,1}\;=\;4.9$ kHz corresponding to the natural frequency observed in the modal analysis test, Figure 6. On the other hand, the peak of the out-of-plane frequency-response curve appears at 4.3 kHz. We also note that the signal-to-noise ratio of the in-plane frequency-response curve is higher, with the peak reaching 178 mm/s, than that of the out-of-plane curve where the peak is 3 mm/s.

While the peak of the out-of-plane curve can still be resolved accurately, it would be erroneous to conclude that it corresponds to the resonance of the first in-plane bending mode. In fact, the out-of-plane curve drops to a minimum at the correct resonance frequency $f_{i,1}\;=\;4.9$ kH. This is expected since the out-of-plane frequency-response measures spurious motions associated with the in-plane mode. As a result, it drops towards a minimum at the impedance of the in-plane mode approaches a minimum at its resonance, thereby absorbing the energy in the actuator.

**Figure 8:**
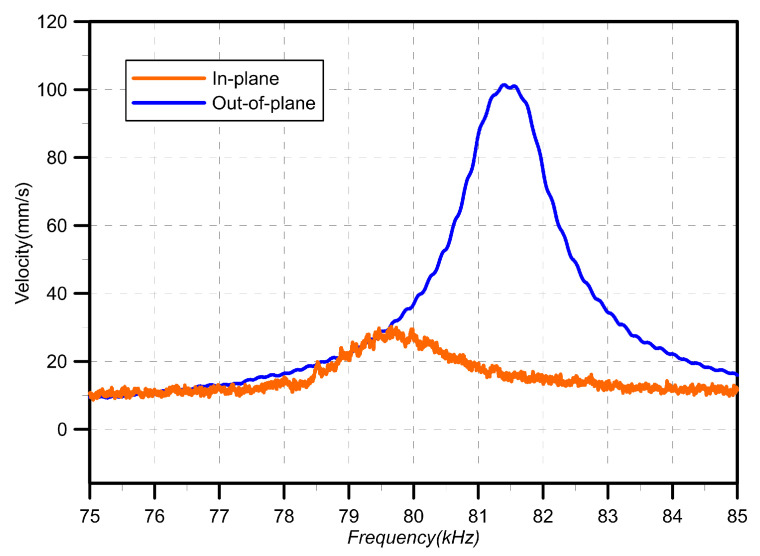
Out-of-plane and in-plane measurements of the frequency-response curves of the actuator under the excitation waveform $V_{o}=7.5$ V as the frequency is swept from 75 kHz to 85 kHz.

The frequency-response curves of the third out-of-plane bending mode under the same excitation waveform were determined by sweeping the signal frequency from 75 kHz to 85 kHz, Figure 8. The velocity peak appears at 81.5 kHz in the out-of-plane curve while the in-plane curve peak is found at 79.7 kHz. Both peaks appear at a higher frequency than that observed in the modal analysis at $f_{o,3}\;=\;75.5$ kHz. The out-of-plane motions of the comb fingers within the electrostatic field imposed by the voltage waver form are responsible for that stiffening effect [16].

The two peaks observed in the out-of-plane and in-plane frequency responses offer competing estimates of the resonant frequency. One of them only is correct since they are two observations for the same motions of the third out-of-plane bending mode. The in-plane response, in this case, is composed of a forced response to the comb-drive in-plane excitation and spurious motions associated with the out-of-plane mode. Therefore, we conclude that the correct natural frequency under this voltage waveform is $f_{o,3}\;=\;81.5$ kHz. The fact that the signal-to-noise ratio of the out-of-plane frequency-response curve, where the peak reaches 101 mm/s, is higher than that of the in-plane frequency-response curve, where the peak reaches only 28 mm/s, further confirms this fact.

## 4. Conclusions

We demonstrated an experimental technique to measure in-plane motions of MEMS using a typical out-of-plane (single-axis) LDV. It employs a tilt stage to align the sidewalls of MEMS actuators with the incident laser beam. We demonstrated our technique successfully in measuring the in-plane modal response and frequency response of a comb-drive actuator.

Furthermore, we compared the validity of using in-plane and out-of-plane measurement techniques in detecting predominantly planar modes of response. We found that measuring the response of a mode in a plane where it is not active leads to inferior and misleading results. The motions associated with a planar mode that appear outside its dominant plan are spurious, related to fabrication or actuation misalignment. As a result, they are not only small compared to those occurring in the primary plane of motion, but also approach a minimum as the frequency of excitation approaches the natural frequency of the mode, thereby concentrating the actuator response (energy) along its dominant plane of motion. Therefore, predominantly planar modes should only be observed in their dominant plane. The measurement technique proposed here allows us to achieve that regardless of the plane of motion.

The main drawback of this technique is that it cannot measure in-plane and out-of-plane motions simultaneously. Furthermore, measurable displacements are limited to values smaller than the sidewall height. However, this is not a significant limitation in most MEMS where displacements are typically small compared to the dimensions of the structures.

Typically, the optimal tilt angle is in the range of $60^{\circ }$ to $90^{\circ }$. In fact, a tilt angle of $90^{\circ }$ can be used to acquire in-plane measurements with a traditional on-axis setup. However, this may not be possible due to the presence of other devices on the substrate blocking the optical path to the DUT walls. On the other hand, angles lower than $90^{\circ }$ scatter the laser beam away from the photodetector and result in its dissipation. As the angle deviation from $90^{\circ }$ increases, the returned laser power drops eventually precluding measurement once it drops below the photodetector threshold. Therefore, the user should use angles larger than $60^{\circ }$ and approach $90^{\circ }$ as much as possible. While lower angles may be possible, they are not recommended since they increase the noise floor due to a lower signal-to-noise ratio. Even at the optimal tilt angle, the noise floor of in-plane measurements is typically higher than that of out-of-plane measurements.
